# Quantitative FRET Microscopy Reveals a Crucial Role of Cytoskeleton in Promoting PI(4,5)P_2_ Confinement

**DOI:** 10.3390/ijms222111727

**Published:** 2021-10-29

**Authors:** Maria J. Sarmento, Luís Borges-Araújo, Sandra N. Pinto, Nuno Bernardes, Joana C. Ricardo, Ana Coutinho, Manuel Prieto, Fábio Fernandes

**Affiliations:** 1 Centro de Química-Física Molecular and Institute of Nanoscience and Nanotechnology, Instituto Superior Técnico, University of Lisbon, 1049-001 Lisbon, Portugal; 2 IBB-Institute for Bioengineering and Biosciences, Instituto Superior Técnico, University of Lisbon, 1049-001 Lisbon, Portugal; lpborgesaraujo@tecnico.ulisboa.pt (L.B.-A.); sandrapinto@ist.utl.pt (S.N.P.); nuno.bernardes@tecnico.ulisboa.pt (N.B.); joana.ricardo@jh-inst.cas.cz (J.C.R.); ana.coutinho@tecnico.ulisboa.pt (A.C.); manuel.prieto@tecnico.ulisboa.pt (M.P.); 3 Associate Laboratory i4HB—Institute for Health and Bioeconomy at Instituto Superior Técnico, Universidade de Lisboa, Av. Rovisco Pais, 1049-001 Lisbon, Portugal; 4 Departamento de Química e Bioquímica, Faculty of Sciences, University of Lisbon, 1749-016 Lisbon, Portugal; 5 Department of Bioengineering, Instituto Superior Técnico, University of Lisbon, 1049-001 Lisbon, Portugal

**Keywords:** PI(4,5)P_2_, PH domains, membrane organization, membrane domains, FRET microscopy

## Abstract

Phosphatidylinositol 4,5-bisphosphate (PI(4,5)P_2_) is an essential plasma membrane component involved in several cellular functions, including membrane trafficking and cytoskeleton organization. This function multiplicity is partially achieved through a dynamic spatiotemporal organization of PI(4,5)P_2_ within the membrane. Here, we use a Förster resonance energy transfer (FRET) approach to quantitatively assess the extent of PI(4,5)P_2_ confinement within the plasma membrane. This methodology relies on the rigorous evaluation of the dependence of absolute FRET efficiencies between pleckstrin homology domains (PH_PLCδ_) fused with fluorescent proteins and their average fluorescence intensity at the membrane. PI(4,5)P_2_ is found to be significantly compartmentalized at the plasma membrane of HeLa cells, and these clusters are not cholesterol-dependent, suggesting that membrane rafts are not involved in the formation of these nanodomains. On the other hand, upon inhibition of actin polymerization, compartmentalization of PI(4,5)P_2_ is almost entirely eliminated, showing that the cytoskeleton network is the critical component responsible for the formation of nanoscale PI(4,5)P_2_ domains in HeLa cells.

## 1. Introduction

PI(4,5)P_2_ is the most abundant polyphosphoinositide in the inner leaflet of the plasma membrane of mammalian cells (~1 mol%) [1], and is crucial to a multitude of cellular processes, including membrane trafficking, signal transduction, ion channel function, and cytoskeleton dynamics [2]. Protein-induced clustering of PI(4,5)P_2_ has been shown to occur even in membrane model systems [3,4]. Similarly, divalent cations, such as Mg^2+^ and Ca^2+^, were also shown to induce clustering of PI(4,5)P_2_ in liposomes [5,6,7,8,9]. 

Enrichment of PI(4,5)P_2_ within large (μm-sized) plasma membrane patches was already observed through confocal microscopy of PI(4,5)P_2_-binding domains and antibodies [10,11]. These patches colocalized with regions of increased exocytic activity, suggesting that these μm-sized PI(4,5)P_2_ clusters are associated with specialized endocytic/exocytic structures [10,11]. PI(4,5)P_2_-enriched plasma membrane patches (PRMPs) of similar dimensions were also observed in focal adhesion points and sites of extensive membrane ruffling [12,13,14]. 

PI(4,5)P_2_ confinement in the plasma membrane has been confirmed through different methods, including super-resolution fluorescence imaging of pleckstrin homology domains fused with fluorescent proteins (PH_PLCδ_-FP) [15], anti-PI(4,5)P_2_ antibodies [16], and PI(4,5)P_2_ fluorescent analogues [17,18]. Sphingomyelin-dependent nanoscale clustering of PI(4,5)P_2_ was also proposed in HeLa cells, suggesting association to membrane rafts in the outer leaflet of the plasma membrane [19]. Recently, compartmentalization of PI(4,5)P_2_ metabolism into plasma membrane/liquid ordered/raft domains was suggested [20]. Additionally, pools of clustered PI(4,5)P_2_ were detected by electron microscopy (EM), associated with caveolae and the clathrin-coated pit in human fibroblasts and mouse smooth muscle cells [21].

Nevertheless, in undifferentiated areas of the membrane and at the nanoscale, the presence of PI(4,5)P_2_ clusters or domains is not universally observed. In a recent study, single-molecule super-resolution imaging of live insulin-secreting INS-1 cells detected no significant nanoscale PI(4,5)P_2_ clustering and only 200–500 nm sparse patches of moderately increased PI(4,5)P_2_ concentration were observed [22]. Another EM study also proposed a homogeneous distribution of PI(4,5)P_2_ in HEK293 cells [23]. 

The observation of PI(4,5)P_2_ clusters through standard optical imaging techniques is challenging and some of the reported structures of this type are likely the result of artefacts, as described elsewhere [23,24,25]. In this context, FRET is particularly powerful for the characterization of the nanoscale organization of biomembranes [26] and its application to live cell imaging is relatively straightforward and free of the artefacts noted above. FRET imaging with PH_PLCδ_-FPs has in fact been used to monitor changes in PI(4,5)P_2_ content at the plasma membrane [23,27]. The lateral diffusion of PH_PLCδ_-FPs is comparable to that of PI(4,5)P_2_ [28,29], and their distribution mirrors that of fluorescently labelled PI(4,5)P_2_ [14]. Hence, PH_PLCδ_-FPs are well suited to monitor PI(4,5)P_2_ dynamics in live cells. FRET studies using PH_PLCδ_-FP domains focused on recovering qualitative information regarding kinetic changes of PI(4,5)P_2_ levels within the plasma membrane, reflecting variations in the activity of the enzymes associated with the metabolism of this phospholipid [30,31,32]. The main challenge associated to this method is the difficulty in interpreting FRET efficiency (*E*_FRET_) values. In fact, since FRET takes place between non-interacting proteins (so-called “bystander FRET”), it is directly dependent on acceptor expression levels [33,34], and no quantitative information on PI(4,5)P_2_ organization is recovered from these measurements. Here, we make use of a FRET imaging methodology based on the analysis of the dependence of *E*_FRET_ with acceptor PH_PLCδ_-EYFP fluorescence intensity (I_F (PH-EYFP)_) in the plasma membrane of different cell types. 

Previous studies have showed that the FRET efficiencies between non-interacting proteins in membranes are well described by available theoretical models for FRET in two dimensions [35,36]. The analysis of *E*_FRET_ vs. I_F (PH-EYFP)_ profiles, in the context of existing analytical solutions for the problem of FRET in a plane, is expected to allow for the estimation of average confinement of PH_PLCδ_ domains in the plasma membrane. The robustness of the method is confirmed through the analysis of PH confinement using two different FRET pairs. This strategy was used to address the impact of raft-like membrane patches and the actin cytoskeleton on the organization of PI(4,5)P_2_ confinement in flat undifferentiated regions of the plasma membrane of HeLa cells.

## 2. Results

### 2.1. 2D FRET between Non-Compartmentalized Proteins Shows a Linear Dependence with Acceptor Concentration in the Low FRET Range

While measurements of FRET between PH_PLCδ_-FP domains have been successfully employed to monitor fluctuations of PI(4,5)P_2_ levels in the plasma membrane [23], these measurements fail in quantifying the extent of PI(4,5)P_2_ compartmentalization. In fact, FRET between non-interacting partners, such as observed for PH_PLCδ_-FP domains within the plasma membrane, is heavily dependent on the concentration of acceptors in the vicinity of donors [36]. As a result, in a FRET experiment employing PH_PLCδ_-FP domains, results are intrinsically associated to expression levels of the PH_PLCδ_-FP acceptor and no quantitative information regarding the distribution of PI(4,5)P_2_ can be recovered from the measurement of an isolated *E*_FRET_ value. On the other hand, donor concentrations have no impact on FRET efficiencies, and levels of PH_PLCδ_-FP donor do not need to be controlled.

For this work, the donor–acceptor FRET pairs chosen were CFP/YFP, and mTurquoise/YFP. Both of these fluorescence protein pairs demonstrate considerable spectral overlap between donor emission and acceptor absorbance (Figures S1 and S2 of the Supplementary Materials), a necessary condition for FRET.

From analytical models of FRET efficiency between donors and acceptors distributed homogeneously within a two-dimensional plane [37,38], it can be inferred that *E*_FRET_ displays a fully linear relationship with acceptor densities up to a concentration of 8000 molecules/μm^2^ (Figure S3a,b of the Supplementary Materials). This is true for any FRET pair and Förster radius ($R_{0}$) value. Since the average surface density of basal PI(4,5)P_2_ in the inner leaflet of the plasma membrane of an eukaryotic cell was estimated at approximately 4000–5000 molecules/µm^2^ [27,39], we can be confident that FRET efficiency values between PH_PLCδ_-FP domains in the plasma membrane must show a linear dependence on the acceptor fluorescence intensity in case of no compartmentalization.

FRET efficiencies obtained from the analytical model were in full agreement with the Monte Carlo (MC) simulations for FRET in the same systems (Figure S3c). Due to the peptide linker, fluorescent proteins within the PH_PLCδ_-FP bound to the plasma membrane are expected to fluctuate around an average position. MC simulations were then used to estimate the impact that considerable fluctuations in the position of the acceptor EYFP (±15 Å) around an average position would have on *E*_FRET_. The results confirm that the impact of PH_PLCδ_-FP fluctuations around an average position is negligible for this system (Figure S3d), since $R_{0}$ values are considerably greater than the maximum displacement. This validates the two-dimensional approximation for FRET between PH domains in case the plasma membrane exhibits moderate curvature in the measured areas. Modeling of FRET between non-interacting PH domains, both the analytical model and the MC simulations, is described in detail in the Section 1 of the Supplementary Materials. 

**Figure 1:**
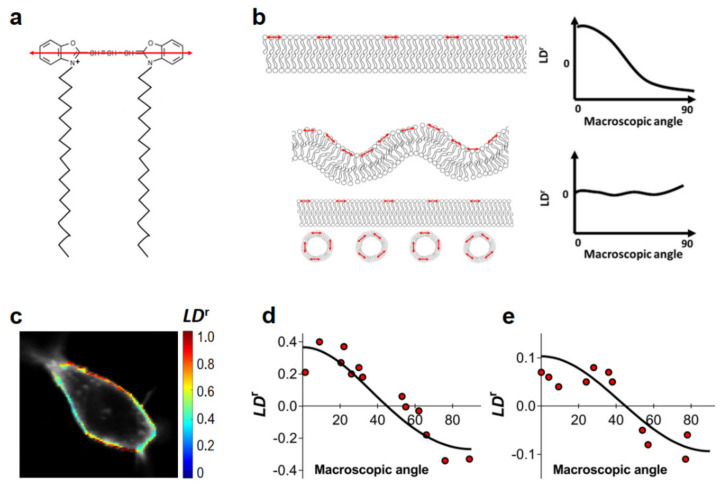
Evaluation of nanoscale ruffling of the plasma membrane. Reduced linear dichroism (*LD*^r^) values of the DiOC18(3) membrane probe in HEK293T and HeLa cells. (**a**) Structure of DiOC18(3). The orientation of the fluorophore’s absorption dipole is shown in red. (**b**) Schematic representation of the impact of different membrane topologies on the recovered *LD*^r^ values of DiOC18(3) when using a polarized excitation source. A planar membrane (top) implies the presence of heavily aligned fluorophores, such that the probability of excitation depends heavily on membrane orientation. In the case of non-flat membranes or in the presence of intracellular vesicles in the immediate vicinity of the plasma membrane (bottom), the orientation of the absorption dipoles of DiOC18(3) is no longer homogeneous and no dependence of *LD*^r^ on macroscopic membrane orientation is expected. Red arrows indicate the orientation of the transition dipole of DiOC18(3). (**c**) *LD*^r^ imaging of DiOC18(3) in a HEK293T cell (false color scale). *LD*^r^ values relative to membrane orientation are shown for HEK293T (**d**) and HeLa (**e**) cells. *LD*^r^ values were determined as described in Section 2 of the Supplementary Materials. Each data point corresponds to ROI in the plasma membrane of a given cell.

In order to confirm that FRET within the plasma membrane of the cells to be used in this study can be approximated by the 2D model, we evaluated their nanoscale ruffling. While at the microscale, it is evident that both HEK293T and HeLa cells exhibit large sections of flat undifferentiated plasma membrane, it is impossible to judge from optical data alone if there is any nanoscale ruffling. Linear dichroism (LD) measurements of the membrane probe DiOC18(3) were previously shown to be highly useful for the characterization of the extent of plasma membrane curvature or ruffling [40,41]. LD describes for a given molecule how the transmittance of linearly polarized light depends on the orientation of polarization and can be used as a tool to detect the presence of nanoscale membrane ruffling. The lipid probe DiOC18(3) binds to cell membranes with the transition dipole oriented parallel to the membrane surface [40] (Figure 1a). Thus, for non-ruffled membranes, the *LD^r^* value of DiOC18(3) should be defined by the angle between the membrane normal and the experiment axis. However, in the presence of plasma membrane ruffles or intracellular vesicles in the vicinity of the plasma membrane, insertion of DiOC18(3) within these structures leads to the randomization of DiOC18(3) orientations. Consequently, *LD^r^* values approach 0 and become independent of the membrane normal angle (Figure 1b). In this way, *LD^r^* values at the plasma membrane are expected to be heavily dependent on the membrane orientation only if little or no ruffling, as well as neighboring intracellular vesicles, are present. For normalization, LD values can be divided by the isotropic absorbance, yielding the reduced linear dichroism (*LD^r^*, see Section 2 of the Supplementary Materials for details). 

We measured *LD^r^* values for DiOC18(3) in both HEK293T and HeLa cells at several regions of interest (ROIs) of the plasma membrane within the equatorial optical section of the cell (Figure 1c). Selected ROIs corresponded to apparently flat undifferentiated areas of the plasma membrane. *LD^r^* values are plotted as a function of the plasma membrane orientation relative to excitation polarization (Figure 1d,e), as determined by visual inspection of confocal images of total DiOC18(3) fluorescence (Figure 1c). Results for the flat undifferentiated sections of the plasma membrane of both HEK293T and HeLa cells are clearly indicative of moderate or absent nanoscale ruffling (Figure 1d,e), as *LD^r^* values of DiOC18(3) are shown to be highly dependent on membrane orientation. In these conditions, we can expect that FRET within the plasma membrane of both HEK293T and HeLa cells to be well described by a 2D approximation.

### 2.2. Bystander FRET in the Absence of Compartmentalization

While the simulations presented in Figure S3 provide a model for the change of FRET efficiency with acceptor density, they cannot be directly compared to experimental data to determine if a given protein FRET pair is clustering. In fact, there is considerable uncertainty regarding several of the simulation parameters, such as the acceptor exclusion radius around the donor, which will have a significant impact on final FRET efficiencies, and is expected to be heavily dictated, not only by steric hindrance, but also by protein dynamics. Additionally, to directly compare the results of theoretical simulations and experimental data obtained in living cells, extensive calibration of both acceptor signal and confocal imaging conditions must be carried out, which can be a significant source of uncertainty for the quantification of compartmentalization.

On the other hand, since acceptor fluorescence intensity values (*I*_*F*(*Acceptor*)_) are directly proportional to the concentrations of that specie, representation of FRET efficiency relative to acceptor fluorescence intensities are also expected to show linearity. In the case of a homogeneous distribution of donors and acceptors, the slope of this relationship ($k_{NC}$, Equation (1)) defines the FRET signature of non-interacting and non-compartmentalized proteins in the plasma membrane and deviations from this value would reflect compartmentalization.

(1) $$E_{\mathrm{FRET}}\left(NC\right)=k_{NC}\cdot I_{F\left(Acceptor\right)}$$

In order to evaluate the FRET dependence with acceptor fluorescence intensity in the absence of compartmentalization, experiments were first carried out with acylated CFP and YFP. Fusion constructs based on the fluorescent proteins and an acylation substrate sequence from the 13 NH_2_-terminal residues of the kinase Lyn were used [42]. The constructs myrpalm-mCFP and myrpalm-mYFP partition readily to the plasma membrane, showing a non-linear dependence of FRET with acceptor density upon clustering of the constructs within Madin-Darby canine kidney (MDCK) cells [42]. In that study, clustering was disrupted by cholesterol extraction, giving rise to a clear linear dependence of FRET efficiency with acceptor density [42]. 

**Figure 2:**
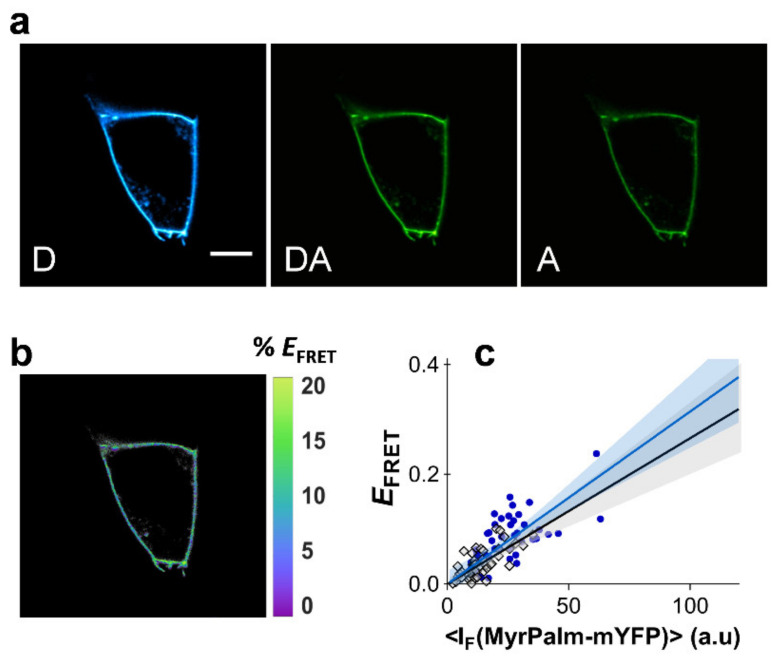
Dependence of FRET efficiency with acceptor intensity. FRET microscopy of HEK293T and HeLa cells co-transfected with myrpalm-mCFP and myrpalm-mYFP. (**a**) Example of confocal data acquired according to the three-filter cube method in HEK293T cells: D—donor channel, DA—FRET channel, A—acceptor channel. Scale bar = 5 μm. (**b**) FRET efficiency image. (**c**) Dependence of *E*_FRET_ with myrpalm-mYFP fluorescence intensity for HEK293T (blue circles) and HeLa cells (gray diamonds). Each data point corresponds to the FRET signal at a segment of the plasma membrane of an individual cell (at equatorial optical sections). Lines represent the global least-squares fit of Equation (1) to both data sets, and the corresponding 95% confidence intervals are shown as shaded areas (see Section 4 of the Supplementary Materials).

It is therefore possible to employ the acylated fluorescent proteins to quantify dependence of FRET efficiency with acceptor intensity. In order to achieve this, myrpalm-mCFP and myrpalm-mYFP were co-expressed in both HEK293T and HeLa cells and FRET was measured using the three-filter cube FRET microscopy approach (Figure 2a,b, see Section 4 for details) [43]. The areas of the plasma membrane selected for FRET analysis were only flat undifferentiated regions, where no heterogeneities were visible in the confocal microscope. *E*_FRET_ values were determined as a function of myrpalm-mYFP fluorescence intensity (Figure 2c). Each datum point corresponds to the FRET efficiency at a segment of the plasma membrane of an individual cell (at equatorial optical sections), and each cell is only measured once in a representative area. The recovered FRET efficiencies show a markedly linear dependence with myrpalm-mYFP fluorescence intensity, for both cell types (Figure 2c). Typically, unconstrained linear regression of the data recovered very small intercept values (Section 3 of the Supplementary Materials). A linear regression model without intercept was chosen and fitted to the data as this represents a more realistic model and avoids overparameterization of the fitting procedure, which could obscure the interpretation of results. Additionally, both cell types exhibit almost identical slopes for the relationship between *E*_FRET_ and myrpalm-mYFP intensity. The slope is also not affected by cholesterol extraction with methyl-β-cyclodextrin (MβCD, Figure S5; Section 5 of the Supplementary Materials). These results confirm a non-clustered distribution of the fluorescent proteins, and the resulting average slope for these experiments was taken to represent $k_{NC}$ ($k_{NC}$ = 2.04 × 10^−4^ ± 3.22 × 10^−5^), as described in Equation (1).

The $k_{NC}$ for another FRET pair can be estimated considering the differences in Förster radiuses. The $R_{0}$ for CFP/YFP is 49 Å, while that of mturquoise/YFP is 56 Å. The difference in the slope of the FRET relationship with acceptor fluorescence intensity is, hence, 61%, according to the analytical models for FRET efficiency [37,38], and the $k_{NC}$ value for mturquoise/YFP, calculated from the $k_{NC}$ value obtained above, is 3.64 × 10^−4^. Deviations from these values will then be proof of compartmentalization and the results will be described by

(2) $$E_{\mathrm{FRET}}=R_{C}\cdot k_{NC}\cdot I_{F\left(Acceptor\right)}$$

where $R_{C}$ is the compartmentalization ratio, and it reflects the average nanoscale concentration increase of acceptor constructs around donors.

### 2.3. Clustering of PI(4,5)P_2_ in HEK293T Cells 

**Figure 3:**
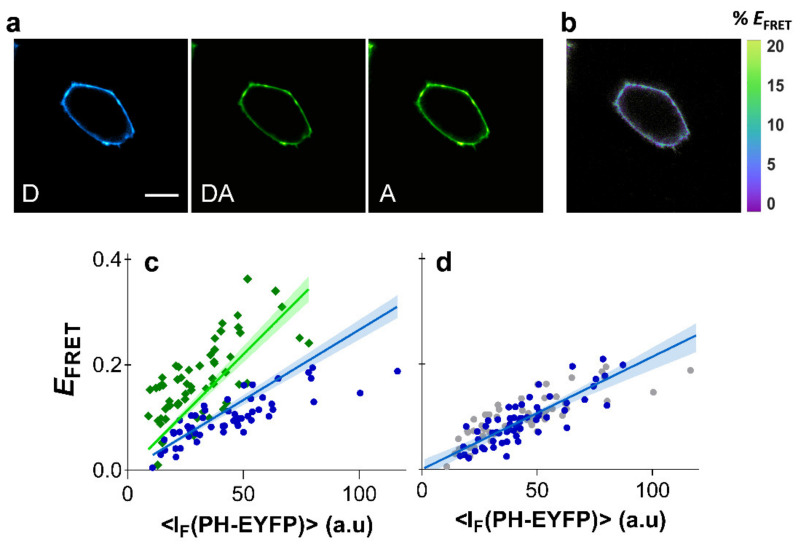
Clustering of PI(4,5)P_2_ in HEK293T cells. FRET microscopy of HEK293T cells co-transfected with PH_PLCδ_-ECFP (or PH_PLCδ_-mTurquoise) and PH_PLCδ_-EYFP. (**a**) Example of confocal data acquired according to the three-filter cube method: D—donor channel, DA—FRET channel, A—acceptor channel. Scale bar = 5 μm. (**b**) FRET efficiency image. (**c**) Dependence of *E*_FRET_ with PH_PLCδ_-EYFP fluorescence intensity using PH_PLCδ_-ECFP (blue) or PH_PLCδ_-mTurquoise (green) as the donor. Each data point corresponds to the FRET signal at a segment of the plasma membrane of an individual cell (at equatorial optical sections). Lines represent the global least-squares fit of Equation (2) to both data sets, and the corresponding 95% confidence intervals are shown as shaded areas. An $R_{C}$ value of 0.844 ± 0.33 was recovered from the global analysis. (**d**) FRET data obtained from cells expressing PH_PLCδ_-ECFP and PH_PLCδ_-EYFP after cholesterol extraction with MβCD (blue) was identical to control cells (grey). Lines represent the least-squares fit of Equation (2) to both data sets, and the corresponding 95% confidence intervals are shown as shaded areas.

PH_PLCδ_-ECFP (or PH_PLCδ_-mTurquoise) and PH_PLCδ_-EYFP were co-expressed in HEK293T cells and FRET was measured in cells exhibiting fluorescence from both constructs. The spectroscopic properties of ECFP and EYFP are almost identical to the mCFP and mYFP, respectively, so that the Förster radii for both pairs are also the same and FRET efficiencies can be readily compared. Expression levels of PH_PLCδ_ domains were minimized to avoid inhibition of PI(4,5)P_2_-mediated cellular functions resulting from considerable competition of PH_PLCδ_ domains with endogenous effectors for PI(4,5)P_2_ binding [44,45,46,47,48]. Expression of PH_PLCδ_ domains at low or moderate levels were previously shown to not drastically impair signaling properties [49]. Under low levels of PH-EYFP expression, recovered FRET efficiencies were naturally moderate, and were found to be <25% for PH_PLCδ_-ECFP (Figure 3).

Results for both FRET pairs were globally fitted with Equation (2), using the previously obtained values for $k_{NC}$ for each FRET pair. Global analysis of FRET data from PH_PLCδ_-ECFP/PH_PLCδ_-EYFP and PH_PLCδ_-mTurquoise/PH_PLCδ_-EYFP was carried out with a shared $R_{C}$ parameter. The robustness and validity of the method shown here is confirmed as data from both FRET pairs were well fitted with a $R_{C}$ value of 0.844 ± 0.33 (Figure 3c), reflecting a general absence of PI(4,5)P_2_ clustering in HEK293T cells.

A previous study using the PH_PLCδ_-ECFP/PH_PLCδ_-EYFP constructs had already shown that FRET efficiency was insensitive to cholesterol extraction in HEK293T cells [23]. Here, efficient plasma membrane depletion of cholesterol in HEK293T cells was confirmed by measurements with the membrane probe Laurdan, whose fluorescence spectrum is sensitive to changes in membrane order (see Section 6 of the Supplementary Materials for details). Significant shifts of Laurdan emission spectrum were identified through generalized polarization (*GP*) measurements after cholesterol extraction with MβCD (Figure S6a). We confirmed that cholesterol extraction from the plasma membrane of HEK293T cells results in identical *E*_FRET_ profiles (Figure 3d). These results confirm that cholesterol is not a modulator of PI(4,5)P_2_ organization in HEK293T cells, and that PI(4,5)P_2_ is not clustered or enriched within plasma membrane raft-like patches of these cells.

### 2.4. Clustering of PI(4,5)P_2_ in HeLa Cells

**Figure 4:**
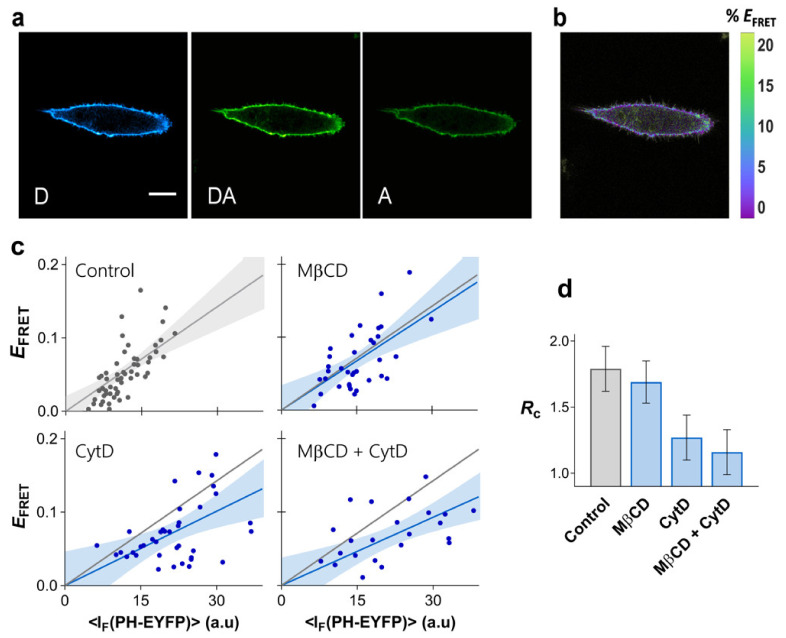
Clustering of PI(4,5)P_2_ in HeLa. FRET microscopy of HeLa cells co-transfected with PH_PLCδ_-ECFP and PH_PLCδ_-EYFP. (**a**) Example of confocal data acquired according to the three-filter cube method: D—donor channel, DA—FRET channel, A—acceptor channel. Scale bar = 5 μm. (**b**) FRET efficiency image. (**c**) Dependence of *E*_FRET_ on PH_PLCδ_-EYFP fluorescence intensity. Each data point corresponds to the FRET signal at a segment of the plasma membrane of an individual cell (at equatorial optical sections). The lines represent the least-squares fit of Equation (2) to the data sets and the corresponding 95% confidence intervals are shown as the shaded areas. FRET was measured on unperturbed cells (control), and on cells exposed to MβCD for cholesterol extraction, or to CytD for disruption of the cytoskeleton. A combined MβCD + CytD treatment was also carried out. The grey line is the fit to the FRET data in the absence of cytoskeleton disruption and is shown for comparison. (**d**) $R_{C}$ values obtained for PH_PLCδ_-ECFP/PH_PLCδ_-EYFP (±SE).

While for HEK293T cells, no evidence existed in the literature for PI(4,5)P_2_ nanoscale compartmentalization, in HeLa cells, nanoscale PI(4,5)P_2_ clustering, or domain enrichment has been reported [19]. FRET imaging data for HeLa cells expressing PH_PLCδ_-ECFP and PH_PLCδ_-EYFP is shown on Figure 4. Expression levels of PH_PLCδ_-EYFP in HeLa cells were considerably lower than in HEK293T cells. However, the FRET efficiency profile obtained for HeLa cells shows that PH_PLCδ_ domains in these cells are considerably more clustered than in HEK293T cells, with a recovered $R_{C}$ = 1.79 ± 0.17 (Figure 4c,d).

### 2.5. Determinants of PI-(4,5)P_2_ Clustering in HeLa Cells

PI(4,5)P_2_ clustering has been associated with many cellular components and functions. Particularly, the possible presence of membrane rafts enriched in cholesterol and sphingomyelin at the plasma membrane of eukaryotic cells, as well as the interaction with the cortical cytoskeleton, have been often suggested to promote PI(4,5)P_2_ compartmentalization [11,12,13,19,21,50,51,52,53,54].

In order to evaluate the importance of the cortical cytoskeleton for PI(4,5)P_2_ nanoscale lateral organization, actin polymerization in HeLa cells was inhibited with cytochalasin D (CytD) (Figure S7, see Section 7 of the Supplementary Materials for details). The resulting *E*_FRET_ profile is presented in Figure 4c, together with the recovered $R_{C}$ (1.16 ± 0.18, Figure 4d). Disruption of the actin cytoskeleton has a dramatic impact in the FRET profile, with the PH_PLCδ_ domains being significantly less compartmentalized after treatment. These results confirm the crucial role of the cortical cytoskeleton in defining PI(4,5)P_2_ organization in the plasma membrane.

While the contribution of raft-like membranes for PI(4,5)P_2_ organization in the plasma membrane of HEK293T cells was ruled out, reports have suggested that sphingomyelin-rich domains are critical for compartmentalization of PI(4,5)P_2_ in HeLa cells [19]. The FRET profile for PH_PLCδ_-ECFP/PH_PLCδ_-EYFP was measured in HeLa cells after cholesterol extraction with MβCD in order to evaluate the role of plasma membrane raft-like domains in the organization of PI(4,5)P_2_ in these cells (Figure 4c). Efficient cholesterol removal was confirmed through Laurdan *GP* measurements (Figure S5b). As observed before for HEK293T, cholesterol levels have no significant impact on the confinement of PH_PLCδ_ domains in HeLa cells ($R_{C}$ = 1.55 ± 0.17, Figure 4c,d). Combined cholesterol removal and CytD treatment induced no further disruption of PI(4,5)P_2_ clustering than CytD treatment alone ($R_{C}$ = 1.07 ± 0.19, Figure 4c,d). Thus, compartmentalization of PI(4,5)P_2_ in HeLa cells is not associated with outer leaflet raft-like domains or any other structure dependent on cholesterol levels.

## 3. Discussion

Here, we demonstrate that it is possible to use FRET microscopy to quantify nanoscale confinement of pleckstrin homology domains. The robustness of the methodology is confirmed through global analysis of FRET data using two different donor–acceptor pairs, with considerably different Förster radii. The almost fully linear relationship between FRET efficiencies and PH_PLCδ_-EYFP fluorescence intensities suggest that overexpression of PH_PLCδ_ domains in this concentration range does not perturb PI(4,5)P_2_ clustering significantly. In fact, in case the levels of PH_PLCδ_ domains employed here were sufficient to alter PI(4,5)P_2_ organization to a significant extent, a change in the *E*_FRET_ vs. PH_PLCδ_-EYFP profile would be expected. 

It should be noted that the results obtained for PI(4,5)P_2_ compartmentalization refer to PI(4,5)P_2_ molecules bound to PH_PLCδ_ domains, and not free phosphoinositides. In this way, the high levels of compartmentalization determined suggest that PH_PLCδ_ domains are not highly effective in sequestering PI(4,5)P_2_ out from enriched domains. A significant fraction (~2/3) of PI(4,5)P_2_ in the plasma membrane is expected to be bound to membrane proteins [28]. A pool of these PI(4,5)P_2_ molecules is expected to be very tightly bound to protein partners, and not fully available for interaction with PH_PLCδ_ domains. This pool of PI(4,5)P_2_ is not probed by this methodology, which is only sensitive to the available PI(4,5)P_2_ population.

Acylated fluorescent proteins were previously shown to cluster in MDCK cells, and that clustering was disrupted through cholesterol extraction [42]. In both cell types employed here (HEK293T and HeLa), the FRET profiles of myrpalm-mCFP/myrpalm-YFP were identical and were insensitive to cholesterol extraction. These results are strongly supportive of a homogeneous distribution of these constructs in the plasma membrane of these cells, and the corresponding slope was taken as corresponding to the FRET signature of non-interacting and non-compartmentalized proteins in the plasma membrane ($k_{NC}$). When using PH_PLCδ_-FP domains, increases in the slope describing the relationship between FRET efficiency and acceptor intensity were then associated to a compartmentalization ratio ($R_{C}$), reflecting the average increase of acceptors around donors.

For HEK293T cells, our results show that PH_PLCδ_ domains exhibit a close to homogeneous distribution, reflecting an absence of clustering of PI(4,5)P_2_. PH_PLCδ_-ECFP and PH_PLCδ_-EYFP were tagged with fluorescent proteins lacking the A206K mutation, which prevents dimerization at high concentrations [42]. The presence of dimerization in the plasma membrane would add great complexity to the analysis presented here. However, the FRET profiles obtained with these proteins was identical to that obtained with the monomeric acylated fluorescent proteins, showing that no significant oligomerization occurs at these expression levels. This observation is also supported by the fact that the FRET profiles of PH_PLCδ_-ECFP/PH_PLCδ_-EYFP and PH_PLCδ_-mTurquoise/PH_PLCδ_-EYFP can be fitted with similar compartmentalization ratios (Figure 3c), as mTurquoise is monomeric.

Our results show that for HeLa cells, the intensity of PH_PLCδ_-EYFP domains in the nanoscale vicinity of PH-donor molecules was 1.79 higher than that observed for acylated proteins, confirming that nanodomains enriched in PI(4,5)P_2_ are present in these cells.

Interestingly, cholesterol concentration has no impact on FRET profiles, proving that the observed nanodomains are not associated with raft-like membrane patches, unlike what was observed for other systems [21,52]. In fact, the enrichment of the polyunsaturated PI(4,5)P_2_ within highly ordered domains is puzzling, since this lipid has been shown to prefer inclusion within disordered phases [7], and membrane rafts are believed to occur only at the outer leaflet of the plasma membrane, while PI(4,5)P_2_ is in the inner leaflet. One possible explanation for this phenomenon would be that although PI(4,5)P_2_ is not incorporated within lipid rafts, its microdomains are aligned with them. Our results demonstrate clearly that for the cell lines studied here, there is in fact no association between PI(4,5)P_2_ microdomains and membrane rafts.

On the other hand, the cytoskeleton is shown to be critical for the formation of PI(4,5)P_2_ enriched compartments in HeLa cells, as disruption of actin polymerization results in a distribution of PI(4,5)P_2_ close to homogeneity. Several proteins (e.g., ERM proteins, vinculin, and talin) responsible for anchoring actin filaments to the membrane interact directly with PI(4,5)P_2_ [55], and this lipid is critical for actin polymerization and cytoskeleton adhesion to the plasma membrane [56].

The results shown here confirm that not only PI(4,5)P_2_ is important for cytoskeleton assembly and organization, but that the cytoskeleton actively contributes to the formation of PI(4,5)P_2_-rich nanodomains in the plasma membrane. This is in agreement with the cluster feedback model [57] where the relation between PI(4,5)P_2_, actin-binding proteins, and actin is bidirectional. While the presence of PI(4,5)P_2_ and other membrane components is crucial for signaling the formation of the cortical cytoskeleton, local remodeling of actin filaments is then able to sequester and limit the diffusion of PI(4,5)P_2_ [58], creating PI(4,5)P_2_-rich nanoscale domains, similarly to actin-dependent clustering of other plasma membrane components [57,59,60,61,62,63]. 

The relatively moderate values recovered for average increase in local concentration of PI(4,5)P_2_ suggest that the enrichment of PI(4,5)P_2_ into functional domains is energetically economic, as marginal increases in PI(4,5)P_2_ concentration guarantees function, as suggested before [22]. The recruitment of kinases to membrane domains of restricted diffusion, due to the presence of actin-based fences, could be sufficient to maintain these structures.

## 4. Materials and Methods

### 4.1. Cell Culture and Transfection

HEK239T (RRID:CVCL_0063) and HeLa (RRID:CVCL_0030) cells were purchased from ATCC (Manassas, VA, USA). Cells were maintained at 37 °C with 5% CO_2_ in DMEM supplemented with 10% fetal bovine serum (FBS) and 1% penicillin/streptomycin. Cells were passaged every 3–4 days. The day before transfection, cells were seeded in 8-well µ-slides (Ibidi, Munich, Germany) coated with poly-L-lysine, at a density of 1 × 10^5^ cells/well. Transfection with plasmid DNA (0.5–1.0 µg pDNA/well) was carried out using Lipofectamine2000 (Thermo Fisher Scientific, Waltham, MA, USA) according to the manufacturer’s instructions.

### 4.2. pDNA Constructs

The pcDNA3 plasmids coding for the phospholipase Cδ1 pleckstrin homology (PH_PLCδ_) domain fused to ECFP (PH-ECFP), EYFP (PH-EYFP) [30], and mTurquoise (PH-mTurquoise) [64] were kindly provided by Dr. K. Jalink (The Netherlands Cancer Institute, Amsterdam, The Netherlands) [30]. PH-EYFP-pET28a was obtained from PH-EYFP-pcDNA3. Briefly, the PH-EYFP sequence flanked by BamHI and NotI restriction sites was inserted into a pET28a vector.

Myrpalm-mCFP and myrpalm-mYFP in pcDNA3 plasmids, coding for lipid-modified fluorescent proteins [42], were a kind gift of Dr. R. Tsien (Howard Hughes Medical Institute, University of California, San Diego, CA, USA). The plasmid coding for the EYFP–ECFP tandem construct (linked by a sequence of 17 amino acids, YFP-17aa-CFP) was a kind gift of Dr. M.C. Montoya (Centro Nacional de Investigaciones Oncológicas, Madrid, Spain) [65].

To create the EYFP-mTurquoise tandem construct, EYFP was first amplified from PH-EYFP using the primers 5′-ATTAT AAGCT TATGG TACCG AGCTC GGATCC-3′ and 5′-TTATT GCGGC CGCCG GGAAT TCGGC TTGTA CAGC-3′. The EYFP PCR product and PH-mTurquoise were cut with HindIII and NotI, and ligated, resulting in the EYFP-mTurquoise encoding plasmid. 

All constructs were checked by sequencing analysis.

### 4.3. Fluorescence Linear Dichroism Imaging

The day before imaging, HEK239 or HeLa cells were seeded in 8-well µ-slides (Ibidi, Munich, Germany) coated with poly-L-lysine. Shortly before imaging, cells were incubated with 10 µM 3,3′-dioctadecyloxacarbocyanine perchlorate (DiOC18 (3)) for 30 min at 37 °C. After incubation, cells were washed twice with PBS and imaged immediately on a Leica TCS SP5 (Leica Microsystems CMS GmbH, Mannheim, Germany) inverted confocal microscope (DMI6000). A 63× apochromatic water immersion objective with a NA of 1.2 (Zeiss, Jena, Germany) was used for all experiments, and a Ti:sapphire laser with a pulse frequency of 80 MHz was used for excitation of DiOC18 (3). Fluorescence was recorded using a PMC-100-4 cooled high-speed PMT detection head (Becker & Hickl GmbH, Berlin, Germany) and images were acquired using a Becker & Hickl SPC 830 module.

For fluorescence linear dichroism imaging, cells were focused at the mid-way axial position and images were collected under all combinations (vertical, horizontal) of excitation and detection polarization. Background fluorescence calculated from non-labelled cells was subtracted to all measured combinations of polarizations. *LD*^r^ was determined in a MATLAB (The MathWorks, Natick, MA, USA) environment. All additional details can be found in the Section 2 of the Supplementary Materials.

### 4.4. Three-Filter Cube FRET Microscopy

All measurements were performed on a Leica TCS SP5 (Leica Microsystems CMS GmbH, Mannheim, Germany) inverted confocal microscope (DMI6000). A 63× apochromatic water immersion objective with a NA of 1.2 (Zeiss, Jena, Germany) was used for all experiments as well as an Argon laser for excitation purposes.

The imaging setup and all the theoretical basis regarding the implementation of filter-cube FRET microscopy is thoroughly described elsewhere [43]. Briefly, all cells containing both donor and acceptor have to be sequentially measured in three different channels: (a) the donor channel, where the donor (ECFP/mTurquoise) is directly excited (λ_ex_ = 458 nm) and emission acquisition is performed in the donor emission range (λ_em_ = 465–500 nm); (b) the FRET channel, which comprises the excitation of the donor (λ_ex_ = 458 nm) and the collection of acceptor’s (YFP) emission (λ_em_ = 505–600 nm); and (c) the acceptor channel, where the acceptors are directly excited (λ_ex_ = 496 nm) and the emission is collected in the acceptor emission wavelength range (λ_em_ = 505–600 nm). Cells expressing only donor and only acceptor were imaged for the determination of the spectral bleed through parameters [43,66], while cells expressing a donor–acceptor tandem construct were imaged for determination of the proportionality constant *G*. These control samples were measured daily to account for any minor variations within the imaging setup.

The methodology relies on the determination of the aforementioned *G* factor because it converts the measured sensitized acceptor emission ($F_{c}$) to FRET-quenched donor fluorescence [43], thus allowing the recovery of accurate real *E*_FRET_ values. This *G* factor is constant for a particular fluorophore pair and imaging setup [43] and can be determined using tandem constructs composed of both donor and acceptor connected by a linker. Here, HEK293T cells expressing EYFP–ECFP or EYFP-mTurquoise were used, since the protein expression levels were higher than in HeLa cells. First, FRET imaging was performed, and sensitized emission was obtained as described above [43]. Sequentially, the lifetime of the donor in the absence and presence of acceptor was determined by fluorescence lifetime imaging (see Section 8 of the Supplementary Materials for details), allowing the calculation of the real FRET efficiency of each donor–acceptor tandem construct. The relationship between sensitized emission $F_{c}$ and the FRET efficiency is given by:

(3) $$E_{\mathrm{FRET}}=\frac{F_{c}/G}{I_{dd}+F_{c}/G}$$

where $F_{c}/G$ is the quenched donor emission and $I_{dd}+F_{c}/G$ is the donor emission in the absence of FRET. The *G* factor was then calibrated as the value for which the FRET efficiency obtained from the three-filter cube FRET method was equal to real *E*_FRET_ recovered from FRET-FLIM.

For all FRET microscopy experiments, cells were used for experiments one day after transfection with PH_PLCδ_-FP encoding plasmids. Prior to imaging, the culture medium was replaced with FBS- and penicillin/streptomycin-free DMEM. All images were acquired at a line-scan speed of 100 Hz and a size of 512 × 512 pixels. For each condition, 20–40 cells were measured in multiple days, to ensure reproducibility. Green fluorescent beads (PS-Speck^TM^ Microscope Point Source Kit from Thermo Fisher Scientific, Waltham, MA, USA) were also imaged in the acceptor channel to allow the day-to-day calibration of YFP intensity.

All data analyses were carried out using custom-written software developed in a MATLAB environment (MathWorks, Natick, MA). Background fluorescence calculated from non-transfected cells was subtracted to all measured channels. A ROI at the plasma membrane was chosen in each individual cell, to avoid any major contributions from the cytosolic fraction of the fluorescent proteins. Only flat non-differentiated regions of the plasma membrane, with highly homogenous fluorescence intensity, were selected to avoid measuring FRET efficiencies on areas with some degree of membrane wrinkling [67] or extensive presence of endocytic structures. FRET efficiency was then determined for each pixel within the selected ROI. The resulting values were converted to *E*_FRET_ histograms, which were well fitted by a normal distribution without constraints, of which the mean value was used for subsequent analysis. This procedure is effective in moderating the outliner pixel values with low probabilities.

Further details on the simulations and data analysis, including the determination of the 95% confidence intervals, can be found in Sections 1, 3 and 4 of the Supplementary Materials.

## Data Availability

Not applicable.

## Supplementary Materials

The following are available online at https://www.mdpi.com/article/10.3390/ijms222111727/s1.
