# Supplementary material for: Quantitative FRET Microscopy Reveals a Crucial Role of Cytoskeleton in Promoting PI(4,5)P2 Confinement
Source: Int J Mol Sci. 2021 Oct 29;22(21):11727. doi: 10.3390/ijms222111727 (PMC8583820; doi:10.3390/ijms222111727)
Supplement: Supplementary file 1 [file ijms-22-11727-s001.zip › ijms-1385432-supplementary.pdf]

Supplementary Materials

Quantitative FRET microscopy reveals a crucial role of cytoskeleton in promoting $PI(4,5)P_2$ confinement

Maria J. Sarmento[1,†,*], Luís Borges-Araújo[1,2], Sandra N. Pinto[1,2], Nuno Bernardes[2], Joana Ricardo[1,2], Ana Coutinho[1,2,3], Manuel Prieto[1,2], Fábio Fernandes[1,2,4,*]

[1]Centro de Química-Física Molecular and Institute of Nanoscience and Nanotechnology, Instituto Superior Técnico, University of Lisbon, Portugal.

[2]IBB-Institute for Bioengineering and Biosciences, Instituto Superior Técnico, Universidade de Lisboa, Lisboa, Portugal.

[3]Departamento de Química e Bioquímica, Faculty of Sciences, University of Lisbon, Lisbon, Portugal.

[4]Department of Bioengineering, Instituto Superior Técnico, University of Lisbon, Lisbon, Portugal.

***Correspondence:** fernandesf@tecnico.ulisboa.pt, Tel: (+351) 218 419 219; maria.sarmento@medicina.ulisboa.pt; Tel: (+351) 217 999 476

[†]Current address: Instituto de Medicina Molecular, Faculdade de Medicina, Universidade de Lisboa, Av. Prof. Egas Moniz, 1649-028 Lisbon, Portugal.

1. Modelling of FRET between non-interacting PH domains

1.1. *Choice of donor-acceptor FRET pairs*

Donor-Acceptor FRET pairs were chosen so that significant overlap between donor emission and acceptor absorption spectra takes place as this is a condition to achieve high FRET efficiencies. The extent of this overlap as well as donor quantum yield and acceptor extinction coefficient determine the Förster radius (R_0) for that donor-acceptor FRET pair (see below). CFP-YFP and mturquoise-YFP were chosen since both of these FRET pairs meet the overlap criteria.

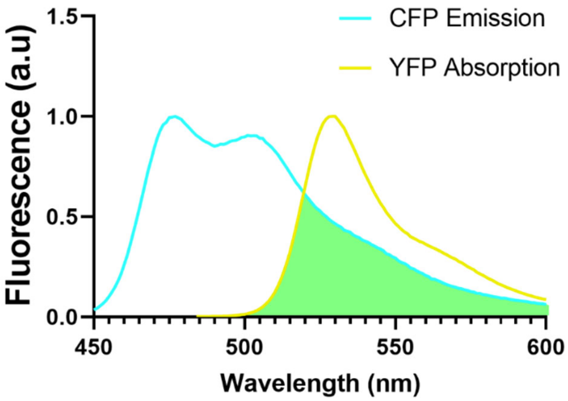

Figure S1. Overlap between CFP emission and YFP absorption spectra. The overlap between the two spectra is shaded green.

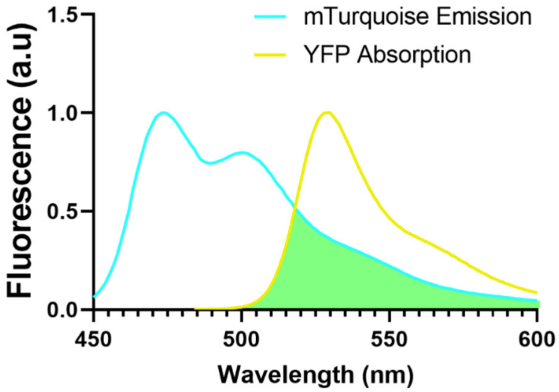

Figure S2. Overlap between mTurquoise emission and YFP absorption spectra. The overlap between the two spectra is shaded green.

1.2. *Analytical model of FRET in a plane*

Since PH domains are only found at the inner leaflet of the plasma membrane, FRET between PH-ECFP and PH-EYFP will occur between populations of fluorescent proteins in close vicinity to the plasma membrane. While linkers between PH domains and fluorescent proteins are sufficient for local flexibility and independent folding of the two domains, they are still relatively small (7-16 amino acids), and fluorescent proteins are not expected to extend significantly away from the plasma membrane. In these conditions, FRET can be modeled for a distribution of donors and acceptors within the same plane (2D). Analytical models for FRET between non-interacting and homogeneously distributed donors and acceptors in a plane describe the dependence of E_{FRET} on $\delta^{D}_{\mathrm{Acceptor}}$:

$$i_{\mathrm{DA}}(t) = \exp\left(-\frac{t}{\tau_0}\right)\exp\left\{-\pi R_0^2 \delta^{D}_{\mathrm{Acceptor}} \gamma\left[\frac{2}{3}\left(\frac{R_0}{R_\mathrm{e}}\right)^6\left(\frac{t}{\tau_0}\right)\right]\left(\frac{t}{\tau_0}\right)^{1/3}\right\}\cdot \\ \cdot\exp\left\{\pi R_\mathrm{e}^2 \delta^{D}_{\mathrm{Acceptor}}\left(1-\exp\left[-\left(\frac{R_0}{R_\mathrm{e}}\right)^6\left(\frac{t}{\tau_0}\right)\right]\right)\right\} \quad (1)$$

where $i_{DA}(t)$ is the fluorescence decay of the donor in the presence of FRET, τ_0 is the fluorescence lifetime of the donor in the absence of acceptors, R_0 is the Förster radius for each specific FRET pair, γ is the incomplete gamma function and R_e is the acceptor exclusion radius around the donor, which is estimated at 47 Å for two fluorescent proteins. $\delta^{D}_{\mathrm{Acceptor}}$ is the numerical concentration of acceptors (molecules per area unit) around donors. From integration of Eq. 1 we get the fluorescence lifetime of the donor in the presence of a given $\delta^{D}_{\mathrm{Acceptor}}$ (τ_{FRET}). This value is then converted to a FRET efficiency by E_{FRET} = 1- $\tau_{\mathrm{FRET}}/\tau_0$.

The R_0 for both the ECFP-EYFP and mTurquoise-EYFP FRET pairs was determined from:

$$R_0 = 0.2108(n^{-4}\Phi_\mathrm{D}\kappa^2 \int f_\mathrm{D}(\lambda)\,\varepsilon_\mathrm{A}(\lambda)^4\,d\lambda\,)^{(\frac{1}{6})} \quad (2)$$

where Φ_D is the donor quantum yield in the absence of acceptors ($\Phi_{\mathrm{D,ECFP}}$ = 0.37; Rizzo et al, *Nat. Biotechnol.* (2004)), $\Phi_{\mathrm{D,mTurquoise}}$ = 0.84; Goedhart et al, *Nat. Methods* (2010)), κ^2 the orientation factor, *n* the refractive index of the medium where FRET takes place, λ the wavelength, f_D the normalized emission spectra of the donor and ε_A is the molar absorption coefficient of the acceptor ($\varepsilon_{\mathrm{A,EYFP}}$= 83400; Cubitt, et al, In *Methods in Cell Biology* (1998)). The numeric factor in Eq. 2 assumes nm units for the wavelength and Å units for R_0. Here, given the flexibility introduced by the linker between PH domains and fluorescent proteins, the κ^2 value was considered to be 2/3, corresponding to the isotropic dynamic regime where randomization of relative orientations of the transition moments of donor and acceptor takes place. Using these parameters, the R_0 was determined to be 49 Å for ECFP/EYFP, and 56 Å for mTurquoise/EYFP. The dependence of FRET efficiency with acceptor concentration according to this model of FRET is shown in Figure S3a,b below for both FRET pairs.

1.3. *MC modeling of FRET in a plane*

The calculation of FRET efficiencies according to Eq. 1 (planar and homogeneous distribution of donors and acceptors) showed no considerable differences relative to the results of MC simulations for random distributions of donors and acceptors within the same plane (Figure S3c). MC simulations were carried out in a Matlab environment. For these simulations, N acceptors are randomly distributed in a planar virtual field of 165 nm x 165 nm, according to a given $\delta_{\mathrm{Acceptor}}$. A donor is included in the center of this area and an acceptor exclusion radius of 47 Å was allowed around the donor. The FRET rate constant (k_{FRET}) between the included donor and each acceptor i in the simulation is calculated according to the distance between donor and acceptor, using the corresponding R_0 value, which in this cased was set to 49 Å:

$$k_{\mathrm{FRET}}^{i} = \frac{1}{\tau_D}\left(\frac{R_0}{d}\right)^6 \quad (3)$$

and E_{FRET} is determined from:

$$E_{\mathrm{FRET}} = \frac{\sum^{N} k_{\mathrm{FRET}}^{i}}{\sum^{N} k_{\mathrm{FRET}}^{i} + \frac{1}{\tau_D}} \quad (4)$$

For each acceptor density, 20,000 simulations were repeated and the average value of E_{FRET} was used. For PH-ECFP, a τ_D of 2.2 ns was measured through FLIM (see the *TCSPC-FLIM* section below).

Due to the peptide linker, fluorescent proteins within the PH-FP bound to the plasma membrane are expected to fluctuate around an average position. In order to evaluate if these fluctuations would result in significantly different FRET efficiencies than the one predicted above for a planar distribution of donors and acceptors, we carried out MC simulations where the acceptors had two possible and equally probable conformations: either within the same plane of the donor or displaced at 15 Å of the donor plane. The results shown in Figure S3d confirm that given the high R_0 values characteristic of fluorescent proteins, fluctuations around an average plane will have a negligible impact on measured E_{FRET} values.

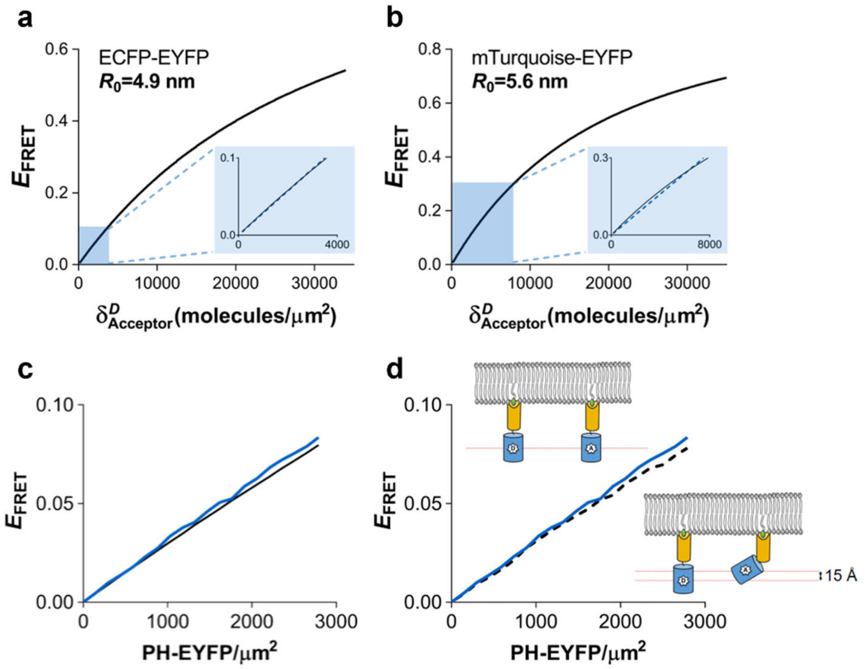

Figure S3. Simulations of FRET between non-compartmentalized PH domains. Dependence of FRET efficiency with PH-EYFP surface density for a homogeneous distribution of membrane-bound and non-interacting (**a**) PH-CFP/PH-EYFP and (**b**) PH-mTurquoise/PH-EYFP, according to the analytical model for FRET efficiency (Fung and L., 1978; Wolber and Hudson, 1979) (Eq. 1).The Förster radius (R_0) of the ECFP/EYFP and mTurquoise/EYFP FRET pairs are 49 and 56 Å, respectively. The analytical solutions for FRET between PH-ECFP/PH-mTurquoise and PH-EYFP within a plane were obtained as described in the text. Since the average surface density of basal PI(4,5)P_2 in the inner plasma membrane of an eukaryotic cell was estimated at approximately 4,000-5,000 molecules/μm^2, simulations were carried out only for concentrations of acceptors (PH-EYFP) below this limit. FRET efficiencies between homogeneously distributed PH-CYFP/mTurquoise and PH-EYFP display a fully linear relationship with acceptor densities in this range. (**c**) Comparison of predicted FRET efficiencies according to MC simulations (blue) and the analytical FRET solution (black) given by Eq. 3, for a homogeneous distribution of acceptors EYFP within the same plane of the donor ECFP. (**d**) MC simulations for random distribution of acceptors EYFP within the same plane of the donor ECFP (blue), and for a distribution of 50% acceptors in the same plane of the donor and 50% acceptors displaced 15 Å from this plane (dashed line). The impact of EYFP fluctuations around an average position is negligible for this system.

---

2. Fluorescence linear dichroism imaging

Linear dichroism (LD) describes how, for a given molecule, the transmittance of linearly polarized light depends on the on the orientation of polarization.

$$LD = A_{\parallel} - A_{\perp} \qquad (5)$$

where $A_{\parallel}$ and $A_{\perp}$ are the absorption of light polarized parallel and perpendicular to a defined experimental axis, respectively. For normalization, this value can be divided by the isotropic absorbance, yielding the reduced linear dichroism (LD^r).

$$LD^r = \frac{A_{\parallel} - A_{\perp}}{A_{\parallel} + 2A_{\perp}} \quad (6)$$

A detailed description of the theory and methodologies involved in *LD* imaging has been described in detail elsewhere (Benninger et al, *Biophys. J.* (2005)). Briefly, a polarized laser is used for excitation in combination with a half-waveplate for changing the polarization of excitation beam (Z and Y, perpendicular to each other). In the detection side, a polarization beamsplitter is used in combination with polarizers to allow the detection of fluorescence at two perpendicular Y and Z polarizations. Four images can then be acquired with all the combinations of excitation and emission polarization: I_{ZZ}, I_{ZY}, I_{YZ}, and I_{YY}. These images are corrected for background intensities and for several factors introduced by the instrumentation, since the laser power is different at each polarization and the instrumentation could transmit one particular polarization over the other (Benninger et al, *Biophys. J.* (2005)). The correction factors for the excitation and detection are E and D_T, respectively, and are obtained from measurements with homogeneous reference solutions of Rhodamine B:

$$D_{\mathrm{T}} = \sqrt{\frac{I_{ZZ} + 2DI_{YZ}}{I_{ZY} + 2DI_{YY}}} \quad (7)$$

$$E = \frac{I_{ZZ} + 2DI_{ZY}}{I_{YY} + 2DI_{YZ}} \quad (8)$$

LD^r images are calculated from the four image acquisitions, and both E and D_T factors:

$$LD^r = \frac{(I_{ZZ} + 2DI_{ZY}) - E(I_{YY} + 2D^{-1}I_{YZ})}{(I_{ZZ} + 2DI_{ZY}) + 2E(I_{YY} + 2D^{-1}I_{YZ})} \quad (9)$$

---

3. Choice of linear regression model

Two possible models could be employed for fitting to the data. These are either a linear regression with an intercept value or a constrained linear regression without intercept. The two models were fitted to the data obtained for FRET between myrPalm-CFP and myrPalmYFP in HEK293T cells (Figure S4)

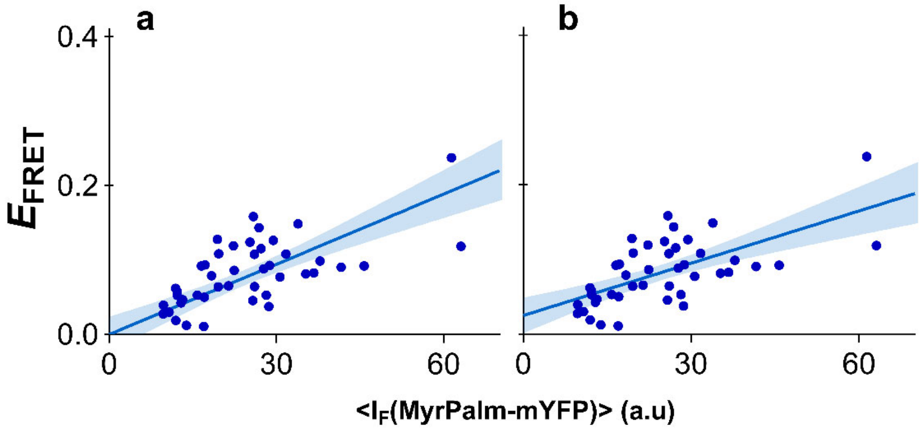

Figure S4: Comparison of constrained (intercept = 0) and unconstrained (intercept = b=0.024850) linear regressions of the data for FRET between myrPalm-CFP and myrPalmYFP in HEK293T cells.

As can be seen form Figure S4, the differences between the two datasets are moderate. In fact the (0, 0) point is at the limit of the 95% confidence interval for the unconstrained linear regression, while the intercept of 0.02485 is also at the limit of the confidence interval of the constrained regression model. While using the regression model with an intercept value naturally does improve the fitting marginally, using it would result in overparameterization of the data, as such model has no physical basis (see analytical solution for FRET in the low range of acceptor concentrations). For these reasons, constrained linear regressions were employed throughout this work.

---

4. Determination of the 95% confidence intervals

95% Confidence Intervals were determined from:

$$CI(95\%) = \hat{E} \mp t_{95\%,(n-2)} S_{yx} \sqrt{\frac{1}{n} + \frac{(\mathrm{x}_i - \overline{x})^2}{\sum_{i=1}^{n}(\mathrm{x}_i - \overline{x})^2}}$$

where $\hat{E}$ is the expected value of *E* for a given value of acceptor fluorescence intensity x_i, $\overline{x}$ is the average of fluorescence intensity values, *n* is the number of measurements and S_{yx} is the standard error of the estimates.

---

5. Effect of cholesterol on the EFRET versus myrpalm-EYFP profile

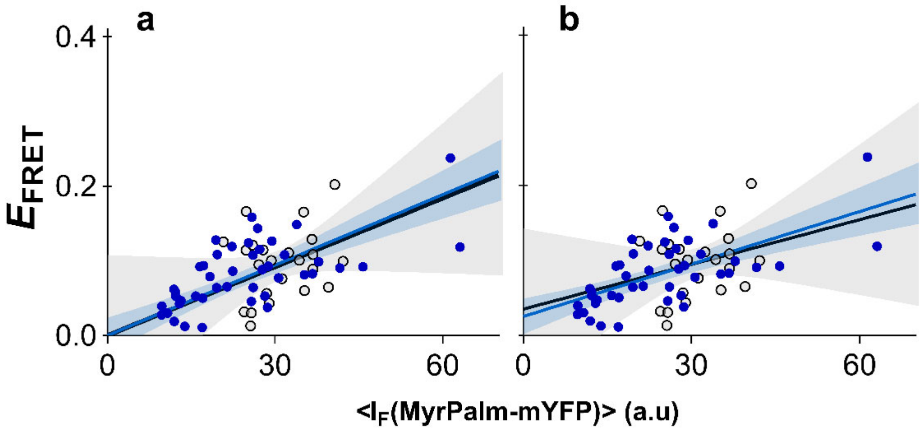

Figure S5. Effect of cholesterol on the dependence of FRET efficiency with acceptor density. FRET microscopy of HEK293T cells co-transfected with myrpalm-mCFP and myrpalm-mYFP. Dependence of E_{FRET} with myrpalm-mYFP fluorescence intensity in the absence (blue circles) and presence (open circles) of MβCD to remove cholesterol from the plasma membrane. Each data point corresponds to the FRET signal at a segment of the plasma membrane of an individual cell (at equatorial optical sections). Lines represent the global least-squares fit of Eq. 2 of the main text to both data sets, and the corresponding 95% confidence intervals are shown as shaded areas. (a) Constrained linear regression (y = Ax), with no intercept value. (b) Non-constrained linear regressions allowing for an intercept value (y = Ax + B). As seen from the examples shown above, when choosing the non-constrained model for fitting the data, for most data sets the value of the intercept B was in fact very close to zero, suggesting that the constrained model is in fact the correct one.

---

6. Cholesterol removal

Cholesterol depletion was accomplished by incubating transfected HEK293T and HeLa cells with MβCD 10 mM for 15 and 60 min, respectively. MβCD was removed from the samples before imaging. Cells were measured right after treatment.

To make sure that cholesterol was removed efficiently from the plasma membrane of both HEK293T and HeLa cells after incubation with MβCD, measurements with a fluorescent probe sensitive to membrane order (6-lauryl-2-dimethylamino-naphtalene, Laurdan, Thermo Fisher Scientific, Waltham, MA, USA) were carried out. The goal was to follow the loss of membrane order that follows cholesterol extraction by monitoring Laurdan fluorescence spectral shifts, using two-photon excitation microscopy. Briefly, cells were incubated with Laurdan 5 µM for 15 minutes at 37 ºC, after incubation with MβCD. Cells were imaged using the same setup described for FLIM (see *TCSPC-FLIM* section below for details). Two-photon excitation was performed at 780 nm, and Laurdan fluorescence emission was collected at 400-460 nm ($I_{400-460}$) and 470-530 nm ($I_{470-530}$). The spectral shifts in Laurdan emission were quantified using the generalized polarization (*GP*) value, defined as:

$$GP = \frac{I_{400-460} - \gamma I_{470-530}}{I_{400-460} + \gamma I_{470-530}} \quad (19)$$

The calibration factor γ was obtained by measuring Laurdan in DMSO, for which the reference *GP* value is known (GP_{DMSO}=0.006; Brewer et al, *Biochim. Biophys. Acta - Biomembr*. (2010)) and using the same experimental conditions used for cell imaging. Both channels were corrected for the background contribution. Images were analyzed using homemade software developed in Matlab environment (Mathworks, Natick, MA). For each condition, at least 8 individual cells were measured. A decrease in Laurdan *GP* is observed after 15 and 60 minutes for HEK293T and HeLa cells, respectively (Figure S5). It indicates a loss in membrane order, which confirms cholesterol removal under the studied conditions.

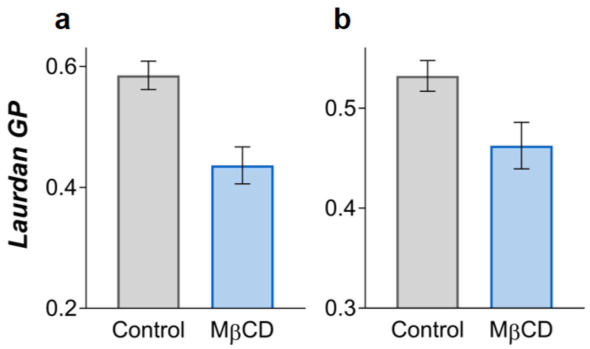

Figure S6. Cholesterol removal confirmation. Laurdan GP measurements performed in (**a**) HEK293T and (**b**) HeLa cells after cholesterol removal with 10 mM MβCD. Following cholesterol removal, cells were incubated with 5 μM Laurdan for 15 (**a**) or 60 (**b**) minutes at 37 ºC. Two-photon excitation was performed at 780 nm, while emission was collected at 400-460 nm and 470-530 nm. GP values were determined using equation 19. The decrease in the GP value is evidence of the decrease in membrane order resulting from cholesterol extraction from the plasma membrane.

7. Cytoskeleton disruption

The disruption of the cytoskeleton was carried out through the incubation of cells with CytD 1 μg/mL for 10 min. ~~CytD was removed from the samples before imaging.~~ The culture medium was exchanged immediately before imaging to remove CytD from the samples. FRET was measured in cells immediately after treatment.

To guarantee that the actin cytoskeleton had been disrupted upon incubation of HeLa cells with CytD, confocal imaging with a cytoskeleton marker was also performed. Briefly, HeLa cells were fixed with 4% paraformaldehyde (PFA) for 10 min at room temperature, before and after treatment with CytD. Cells were then permeabilized with 0.2% Triton X-100 (10 min at room temperature) and incubated with the actin cytoskeleton marker phalloidin-TRICT (Sigma-Aldrich, St. Louis, MO) for 40 min also at room temperature (0.2 μg/mL). Before immobilization, cells were also incubated with 1 μg/mL Hoechst 33342 dye (Thermo Fisher Scientific, Waltham, MA, USA) for 15 min at 37 °C to label the nuclei. Confocal microscopy was carried out by sequentially imaging phalloidin-TRICT and Hoesch. For TRICT imaging, excitation was performed at 514 nm and emission was collected at 530-575 nm. To image the nuclei with the Hoesch dye, two-photon excitation was carried out at 780 nm and emission acquired at 385-485 nm. A

clear disruption of the actin cytoskeleton is observed after incubation of HeLa cells with 1 µg/mL CytD (Figure S6).

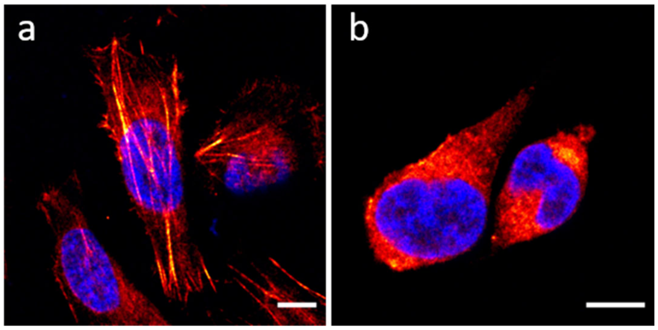

Figure S7. Cytoskeleton disruption confirmation. Confocal imaging of HeLa cells before (**a**) and after actin cytoskeleton disruption with 1 µg/mL CytD (**b**). Cells were fixed with PFA, permeabilized with Triton X-100, and then incubated with phalloidin-TRICT to label the actin filaments (red) and Hoechst 33342 to mark the nuclei (blue). Scale bar: 8 µm. TRICT excitation was performed at 514 nm and two-photon excitation of Hoechst 33342 was accomplished at 780 nm. Emission was collected at 530-575 nm and 385-485 nm, respectively. Incubation with CytD clearly resulted in the disruption of the actin filaments.

---

8. TCSPC-FLIM

Time-correlated single photon counting fluorescence lifetime imaging (TCSPC-FLIM) was performed using a multiphoton Ti:Sapphire laser (Spectra-Physics Mai Tai BB, 710-990 nm, 100 femtoseconds, 80 MHz). A photomultiplier tube was coupled to the X-port of the microscope and the emitted photons were processed by an SPC board that addresses simultaneously the xy location of the collected photons (Becker and Hickl GmbH, PMC-100-4 SPC-830). Moreover, the laser power was adjusted to give an average photon counting rate of ~1x10^4-1x10^5 photons/s and cell images were acquired during ~60 seconds to achieve reasonable photon statistics. Lifetimes of the donor in the absence and presence of acceptor were obtained setting the Ti:Sapphire laser at 780 nm and detecting the emission after passing through a 470-500 nm band-pass filter (for filtering of EYFP fluorescence) and a 700 nm short-pass filter (to block excitation light). The instrumental response function was recorded as excitation light scattered by urea crystals (λ_{ex}= 820 nm).

For FRET efficiency determination, HEK293T cells expressing either only donor (ECFP or mTurquoise) or donor and acceptor (ECFP-EYFP or mTurquoise-EYFP), were used to measure the lifetime of the donor in the absence and presence of acceptors, respectively.

Fluorescence lifetimes were obtained by analysing the fluorescence decays by iterative re-convolution with the IRF using the SPCImage software (Becker & Hickl, Berlin, Germany). The intensity decays were described by:

$$I(t) = \sum_i \alpha_i exp(-t/\tau_i) \qquad (20)$$

where α_i is the normalized amplitude and τ_i is the ith component lifetime. The correspondent amplitude-weighted average lifetime was defined by:

$$\bar{\tau} = \sum_i \alpha_i \tau_i \tag{21}$$

ECFP decay was described by a double exponential while mTurquoise presented a monoexponential decay. The FRET efficiency was given by:

$$E_{\text{FRET}} = 1 - \frac{\bar{\tau}_{DA}}{\bar{\tau}_D} \tag{22}$$

where $\bar{\tau}_D$ and $\bar{\tau}_{DA}$ are the amplitude-weighted average lifetimes of the donor in the absence and presence of acceptors, respectively. For *G* determination purposes (see **Three-filter cube FRET microscopy** in the *Materials and Methods* section of the main text), FRET efficiency values of 0.10 for EYFP-ECFP and 0.45 for EYFP-mTurquoise were recovered by FRET-FLIM.

References

1. Fung, B.K.K.; Stryer, L. Surface Density Determination in Membranes by Fluorescence Energy Transfer. *Biochemistry* **1978**, *17*, 5241–5248.
2. Wolber, P.K.; Hudson, B.S. An analytic solution to the Förster energy transfer problem in two dimensions. *Biophys. J.* **1979**, *28*, 197–210.
